# CMOEP regimen in the treatment of untreated peripheral T-cell lymphoma: a multicenter, single-arm, phase I study

**DOI:** 10.3389/fimmu.2025.1551723

**Published:** 2025-04-11

**Authors:** Jingwei Yu, Xiuhua Sun, Guangxun Gao, Li Yu, Jiesong Wang, Lihua Qiu, Zhengzi Qian, Wei Li, Huilai Zhang

**Affiliations:** ^1^ Department of Lymphoma, Tianjin Medical University Cancer Institute and Hospital, National Clinical Research Center for Cancer, Key Laboratory of Cancer Prevention and Therapy, the Sino-US Center for Lymphoma and Leukemia Research, Tianjin, China; ^2^ Department of Lymphoma of the Head and Neck, The Second Hospital of Dalian Medical University, Dalian, China; ^3^ Department of Hematology, The First Affiliated Hospital, the Air Force Medical University, Xi’an, China; ^4^ Department of Hematology, The Second Affiliated Hospital of Nanchang University, Nanchang, China; ^5^ Department of Medical Oncology, Clinical Oncology School of Fujian Medical University, Fujian Cancer Hospital, Fuzhou, China

**Keywords:** peripheral T-cell lymphoma, mitoxantrone hydrochloride liposome, cyclophosphamide, vincristine, etoposide, prednisone

## Abstract

The current first-line treatment for peripheral T-cell lymphoma (PTCL) has a typically poor prognosis. Developing a new regimen is urgently needed. This phase I study evaluated the maximum tolerated dose (MTD), safety, and efficacy of mitoxantrone hydrochloride liposome (Lipo-MIT) plus cyclophosphamide, vincristine, etoposide, and prednisone (COEP) in untreated PTCL. Patients with untreated PTCL were enrolled and received Lipo-MIT (15, 18, and 20 mg/m^2^) following a 3 + 3 dose-escalation design plus standard doses of COEP (750 mg/m^2^ cyclophosphamide, 1.4 mg/m^2^ vincristine, 60 mg/m^2^ etoposide, and 100 mg prednisone) every 3 weeks for 6 cycles. Primary endpoint was MTD; secondary endpoints were safety, overall response rate (ORR), complete response (CR) rate, progression-free survival (PFS), and overall survival (OS). As of the cut-off date (October 29, 2024), 13 patients received the Lipo-MIT plus COEP (CMOEP) regimen. No patients experienced dose-limiting toxicities (DLT); MTD was 20 mg/m^2^ and the recommended phase 2 dose was 18 mg/m^2^. Common ≥grade 3 hematologic toxicities included neutrophil count decreased (76.9%), white blood cell decreased (76.9%), and lymphocyte count decreased (46.2%). Most common ≥grade 3 non-hematologic toxicity was lung infection (15.4%). No deaths due to toxicities were reported. Among 12 patients evaluated for best response, ORR (95% CI, 73.5-100.0), and the CR rate was 66.7% (95% CI, 34.9-90.1). At a median follow-up of 8.5 months, the median PFS and OS were both not reached. The CMOEP regimen had a manageable safety profile and an encouraging clinical efficacy for PTCL patients, which warrants further investigation.

**Clinical trial registration:**

ClinicalTrials.gov, identifier NCT05458180.

## 1 Introduction

Peripheral T-cell lymphoma (PTCL) constitutes a diverse array of malignancies originating from mature post-thymic T lymphocytes, accounting for 10%-12% of all non-Hodgkin lymphoma (NHL) (1, 2). Clinical outcomes for patients with previously untreated PTCL often depend on the histological subtype but are typically poor, with the current preferred standard first-line therapy option primarily revolving around the chemotherapy regimens CHOP (cyclophosphamide, doxorubicin, vincristine, and prednisone) or a CHOP-like (3). Unfortunately, the standard therapy was beset by a high recurrence incidence and only provided very limited survival advantages, as evidenced by a disappointingly 5-year overall survival (OS) of 30%-40% (4–6). Thus, there is a pressing necessity to investigate and develop innovative first-line therapeutic modalities to improve the prognosis and survival outcomes of PTCL patients.

Adding etoposide to the CHOP regimen (CHOEP) emerged as one of the more successful attempts at improving outcomes for untreated younger PTCL patients, leading to encouraging efficacy with a 5-year OS of 50%-70% (7–9). Nevertheless, a major concern lies in the anthracycline component of the CHOEP regimen, which is associated with serious dose-dependent cardiotoxic side effects that can appreciably increase long-term mortality risks (10, 11). Therefore, continuous research to refine therapeutic approaches is essential to optimize treatment outcomes and minimize potentially life-threatening adverse reactions. In addition, attempts have been made to improve the first-line regimens for PTCL by combining traditional chemotherapy agents with novel agents, such as romidepsin, azacitidine, brentuximab vedotin, and pralatrexate (12–15). However, moving beyond CHOP has proved challenging; even when some agents have been found to improve patient outcomes, but only in specific subtypes (12–14).

Mitoxantrone hydrochloride liposome (Lipo-MIT) is a novel, advanced anthracycline-class antitumor agent. In comparison to conventional anthracyclines, it significantly improves drug stability, enables precise control over drug release, and enhances targeting efficiency, more importantly, its cardiac toxicity is lower (16, 17). Moreover, it has demonstrated encouraging outcomes in pivotal phase II clinical trials for relapsed/refractory PTCL, proving effective in inhibiting tumor growth and prolonged disease control, while maintaining a favorable safety profile (18, 19). Based on the positive clinical evidence, it has been recommended for patients with relapsed/refractory PTCL. Notably, in the context of previously untreated PTCL patients, the application of mitoxantrone hydrochloride liposome remains an area of active exploration. While the recommended Phase 2 dose (RP2D) for Lipo-MIT in combination with cyclophosphamide, vincristine, and prednisone in this patient population has been determined (20), the RP2D of Lipo-MIT when combined with cyclophosphamide, etoposide, vincristine, and prednisone (COEP) regimen for PTCL management has yet to be clarified. Taken together, a phase I dose-escalation clinical trial was initiated to investigate Lipo-MIT in combination with standard doses of COEP (CMOEP) for patients with untreated PTCL, aimed to pinpoint the maximum tolerated dose (MTD) of Lipo-MIT within this combined regimen, assess the safety and efficacy of CMOEP regimen, and thereby lay the groundwork for future clinical trials.

## 2 Materials and methods

### 2.1 Study design

This prospective, single-arm, open-label, multicenter, dose-escalation, phase I study (*ClinicalTrials.gov* identifier NCT05458180) of CMOEP in patients with untreated PTCL was conducted at five centers in China. The cutoff date for safety and activity data was October 29, 2024. This study was approved by the Ethics Committee of the Tianjin Medical University Cancer Institute & Hospital (No. E20220336) and other participating centers, and was performed in accordance to the Declaration of Helsinki, the international standards of good clinical practice, and applicable local laws and regulations. All patients signed written informed consent before any study-related procedure.

### 2.2 Patient eligibility

Eligible patients were 18 to 65 years of age with histologically confirmed PTCL; an Eastern Cooperative Oncology Group (ECOG) performance status score ≤ 1; a life expectancy of ≥ 3 months; had not received previous treatments for PTCL; at least one measurable lesion according to Lugano2014 criteria; and adequate organ function. The key exclusion criteria were hypersensitivity to any of the study drugs or their components; uncontrolled systemic disease; and other malignant tumors. Complete eligibility criteria are listed in the **Supplementary Table S1**.

### 2.3 Procedures

According to the traditional 3 + 3 design, Lipo-MIT was assessed at 3 dose levels (15, 18, 20 mg/m^2^ on day 1), in combination with cyclophosphamide (750 mg/m^2^ on day 1), vincristine (1.4 mg/m^2^ [maximum 2 mg] on day 1), etoposide (60 mg/m^2^ on days 1-3), and prednisone (100 mg on days 1-5) for 6 cycles, with each cycle spanning 3 weeks. Dose-limiting toxicities (DLTs) were monitored during the first treatment cycle and defined as adverse events (AEs) related to the CMOEP regimen that met the predefined criteria based on grading per National Cancer Institute Common Terminology Criteria for Adverse Events (NCI-CTCAE 5.0). DLT included any one of the following: (1) grade 4 neutropenia lasting for >7 days despite the administration of granulocyte-colony stimulating factor; (2) grade 4 thrombocytopenia lasting for >7 days or platelet count ≤10×10^9^/L; (3) grade 4 anemia; (4) grade ≥3 thrombocytopenia with significant bleeding tendency and required blood transfusion; (5) grade ≥3 non-hematologic toxicity, except grade ≥3 neutropenia with fever lasting for ≤7 days after corresponding treatment (including anti-infection, granulocyte colony-stimulating factor, etc.); grade 3 fatigue recovered to grade ≤1 or baseline within ≤7 days; grade 3 loss of appetite recovered to grade ≤1 or baseline within ≤7 days after symptomatic treatment; grade 3 nausea/vomiting/diarrhea/electrolyte disturbances recovered to grade ≤1 or baseline within ≤7 days after symptomatic treatment; Transient grade 3 fever for ≤3 days; grade 3 infection, elevated blood glucose but controllable, insomnia; grade 3 hypertension, which recovered to grade ≤1 or baseline within ≤3 days.

The dose adjustment for Lipo-MIT was graded into two levels: when grade 4 hematologic toxicity or grade 3 non-hematologic toxicity (excluding alopecia) occurred one time or grade 3 hematologic toxicity occurred two times, the dose was reduced to 75% of the starting dose; if further intolerance ensues, a second reduction to 50% of the initial dose was implemented. Even after these two reductions, failure to tolerate the regimen necessitates discontinuing the treatment. If patients continue treatment, they must meet the criteria of neutrophil count ≥1.5×10^9^/L, hemoglobin level ≥80g/L, and platelet count ≥75×10^9^/L, or non-hematological toxicity (excluding alopecia) recovered to grade ≤1 or baseline. Other drug dose adjustments were performed according to drug instructions.

Safety and tolerability were monitored from the time of signing informed consent until the end of follow-up. AEs were graded according to the NCI-CTCAE 5.0. Patients were monitored by vital signs, physical examination, ECOG performance status, laboratory analyses (hematology, biochemistry, urinalysis), and cardiac safety tests at baseline, before every cycle, and at the end of treatment. Tumor responses were evaluated by independent imaging experts according to Lugano 2014 Efficacy Evaluation Criteria, using positron emission tomography-computed tomography (PET-CT) or enhanced CT. Specifically, CT was conducted at baseline, before cycle 3, before cycle 5, and at the end of treatment. PET-CT assessment was administered at baseline, at the end of treatment, and whenever disease progression was suspected or deemed necessary by the investigator.

### 2.4 Endpoints

The primary endpoint was the maximal tolerable dose (MTD), defined as the highest dose at which no more than one of all patients evaluated for toxicity experienced a DLT. If the MTD within the CMOEP regimen was identified, this MTD would be selected as the RP2D. Conversely, if the MTD was not determined, the RP2D was established by the investigators and all centers, based on the safety and efficacy of the study. Secondary endpoints were safety assessed by DLT and treatment-related AEs (TRAEs), overall response rate (ORR), complete response (CR) rate, progression-free survival (PFS), and overall survival (OS). CR rate was defined as the percentage of patients achieving the best overall response of CR. ORR was defined as the proportion of patients with the best overall response of CR or partial response (PR). PFS was defined as the time between the treatment initiation and the first documented disease progression or death of any cause. OS was defined as the time between the treatment initiation and the death of any cause.

### 2.5 Statistical analysis

The sample size for this phase I study was estimated using a typical 3 + 3 dose-escalation design, which included three to six patients in each dose cohort. This estimation was guided by clinical, empirical, and practical considerations specific to this study, without statistical considerations. Safety and survival were assessed in the full analysis set (FAS), including all patients who received at least one dose of study medication and had at least one follow-up record. Tumor response was assessed in patients who received at least one dose of study medication and could be evaluated for response. All data were summarized descriptively due to the phase I exploratory nature of the study. Categorical variables are summarized by percentage and continuous variables are summarized by medians. ORR and CR rate with corresponding 95% confidence interval (CI) were calculated by the Clopper-Pearson method. PFS and OS were estimated using the Kaplan-Meier method and expressed as medians with 95% CI. Statistical analyses were performed using SPSS software (version 25.0).

## 3 Results

### 3.1 Patient characteristics

**Figure 1:**
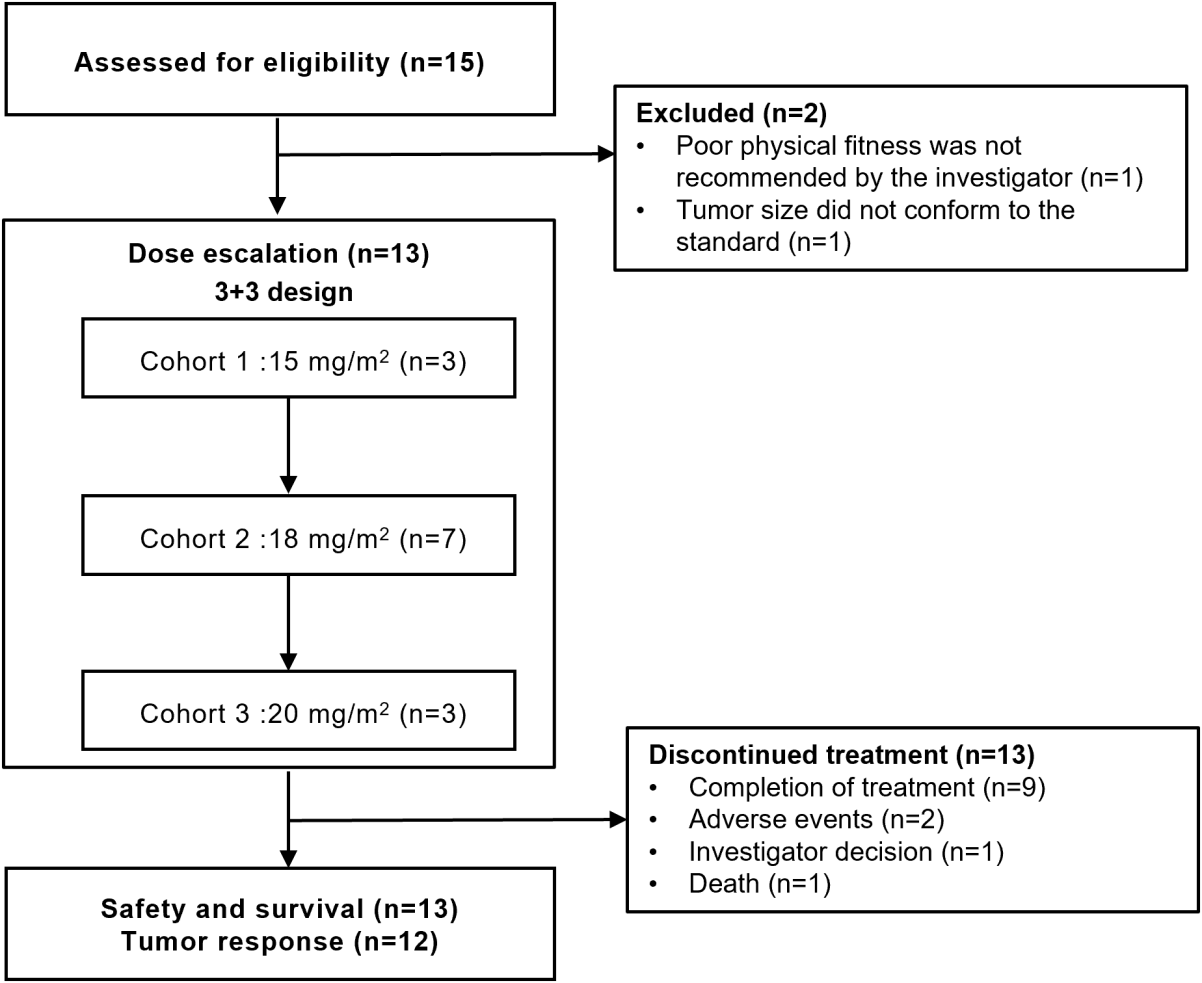
Trial profile.

From August 30, 2022 to March 14, 2024, 15 patients with untreated PTCL were screened and the final 13 were included in this study (**Figure 1**). Patient demographics and baseline characteristics are shown in **Table 1**. The median age of 13 patients was 52 years (range, 19-64), of which 7 (53.8%) were male. The enrolled patients including 4 (30.8%) with peripheral T-cell lymphoma, not otherwise specified (PTCL-NOS), 3 (23.1%) with angioimmunoblastic T-cell lymphoma (AITL), 2 (15.4%) with anaplastic lymphoma kinase (ALK)-negative angiotropic large cell lymphoma (ALK^-^ ALCL), 1 (7.7%) with ALK-positive ALCL (ALK^+^ ALCL), 1 (7.7%) with cutaneous T-cell lymphoma (CTCL), 1 (7.7%) with nodal TFH lymphoma, follicular-type (nTFHL-F), and 1 (7.7%) with monomorphic epitheliotropic intestinal T-cell lymphoma (MEITL). Seven (53.8%) patients were classified as being in Ann Arbor stage IV, only 1 (7.7%) were at high-intermediate risk according to the International Prognostic Index (IPI), and 5 (38.5%) had B symptoms. All patients had an ECOG performance status of 0-1.

**Table 1:** Patient characteristics.

Characteristic	Patients (n=13)
Median age, years (range)	52 (19.0, 64.0)
Sex, male	7 (53.8)
PTCL subtype	
PTCL-NOS	4 (30.8)
AITL	3 (23.1)
ALK^-^ ALCL	2 (15.4)
ALK^+^ ALCL	1 (7.7)
CTCL	1 (7.7)
nTFHL-F	1 (7.7)
MEITL	1 (7.7)
Ann Arbor stage	
Stage I	2 (15.4)
Stage II	2 (15.4)
Stage III	2 (15.4)
Stage IV	7 (53.8)
International prognostic index	
Low risk (0-1)	9 (69.2)
Low-intermediate risk (2)	3 (23.1)
High-intermediate risk (3)	1 (7.7)
ECOG performance status	
0	7 (53.8)
1	6 (46.2)
B symptoms	5 (38.5)
Ki67 status	
<70%	8 (61.5)
≥70%	4 (30.8)
Unknown	1 (7.7)

Data are presented as the median (range) or n (%).

PTCL, peripheral T-cell lymphoma; PTCL-NOS, peripheral T-cell lymphoma, not otherwise specified; AITL, angioimmunoblastic T-cell lymphoma; ALCL, angiotropic large cell lymphoma; CTCL, cutaneous T-cell lymphoma; nTFHL-F, nodal TFH lymphoma, follicular-type; MEITL, monomorphic epitheliotropic intestinal T-cell lymphoma; ECOG, Eastern Cooperative Oncology Group.

### 3.2 DLTs and MTD

All 13 patients underwent treatment with the CMOEP regimen and no DLTs were recorded across the 3 dosing cohorts utilizing Lipo-MIT at respective levels of 15 mg/m^2^, 18 mg/m^2^, and 20 mg/m^2^. Consequently, there was no need for cohort expansion or planned dose escalation beyond 20 mg/m^2^, establishing 20 mg/m^2^ as the MTD within this regimen. Considering both the safety and efficacy, the 18 mg/m² dose emerged as offering comparable therapeutic benefits but with a more favorable safety profile. Therefore, the 18 mg/m² dose was considered as RP2D for subsequent studies.

### 3.3 Safety

All 13 patients who received the CMOEP regimen were assessed for safety. TRAEs of any grade occurred in 12 (92.3%) patients, with the most common hematologic toxicities of any grade being neutrophil count decreased (92.3%), white blood cell decreased (92.3%), anemia (76.9%), lymphocyte count decreased (69.2%), platelet count decreased (61.5%). All patients who received a dose level of 15 mg/m^2^ or 20 mg/m^2^ cohort experienced at least one TRAE and 6 of 7 (85.7%) patients who received a dose level of 18 mg/m^2^ cohort experienced TRAEs of any grade (**Supplementary Table S2**). Grade ≥3 TRAEs of all patients are reported in **Table 2**. Grade ≥3 hematologic toxicities were reported as neutrophil count decreased (76.9%), white blood cell decreased (76.9%), lymphocyte count decreased (46.2%), anemia (38.5%), platelet count decreased (15.4%), and febrile neutropenia (7.7%). Most non-hematologic toxicities were <grade 3, and grade ≥3 included lung infection (15.4%), fatigue (7.7%), and infections (7.7%).

**Table 2:** Treatment-related adverse events (≥grade 3) in the safety population.

TRAEs, ≥grade 3	15 mg/m^2^ (n=3)	18 mg/m^2^ (n=7)	20 mg/m^2^ (n=3)	Total (n=13)
All TRAEs	2 (66.7)	6 (85.7)	3 (100.0)	11 (84.6)
Hematologic				
Neutrophil count decreased	1 (33.3)	6 (85.7)	3 (100.0)	10 (76.9)
White blood cell decreased	2 (66.7)	5 (71.4)	3 (100.0)	10 (76.9)
Lymphocyte count decreased	1 (33.3)	3 (42.9%)	2 (66.7)	6 (46.2)
Anemia	1 (33.3)	3 (42.9)	1 (33.3)	5 (38.5)
Platelet count decreased	0	1 (14.3)	1 (33.3)	2 (15.4)
Febrile neutropenia	1 (33.3)	0	0	1 (7.7)
Non-hematologic				
Lung infection	0	2 (28.6)	0	2 (15.4)
Fatigue	0	0	1 (33.3)	1 (7.7)
Infections	1 (33.3)	0	0	1 (7.7)

Data are presented as n (%).

TRAEs, treatment-related adverse events.

Cardiac toxicities of any grade were reported as palpitation (7.7%), sinus arrhythmia (7.7%), electrocardiogram T wave abnormal (7.7%), and sinus tachycardia (7.7%). No patient experienced cardiac toxicity of grade ≥3. Cardiac biomarkers were assessed in 3 or more patients (**Supplementary Figure S1**, **Supplementary Table S3**). Myocardial troponin T (cTnT) and myocardial troponin I (cTnI) abnormalities were present in the same patient at baseline and the end of treatment. The median brain natriuretic peptide (BNP) levels were stable at 10 pg/mL from the start to the end of the treatment. Throughout the treatment cycle, the left ventricular ejection fraction (LVEF) and high-sensitivity cardiac troponin I (hs-cTnI) levels remained within the normal range, with the median LVEF consistently at 64% before and after treatment. In conclusion, there were no significant changes in these cardiac biomarkers before and after treatment.

Toxicities leading to treatment delay and dose adjustment occurred in 2 (16.7%) and 7 (53.8%) patients, respectively. The toxicities causing treatment delay predominantly comprised grade 3 fatigue, grade 3 platelet count decreased, grade 2-4 white blood cell decreased, grade 3-4 neutrophil count decreased, and grade 2 limb numbness. Meanwhile, dose adjustments were required due to grade 3-4 neutrophil count decreased, white blood cell decreased, fatigue, and infections. One patient discontinued treatment due to grade 4 white blood cell decreased, grade 4 neutrophil count decreased, grade 3 platelet count decreased, and grade 3 anemia. No treatment-related deaths occurred.

### 3.4 Efficacy

**Figure 2:**
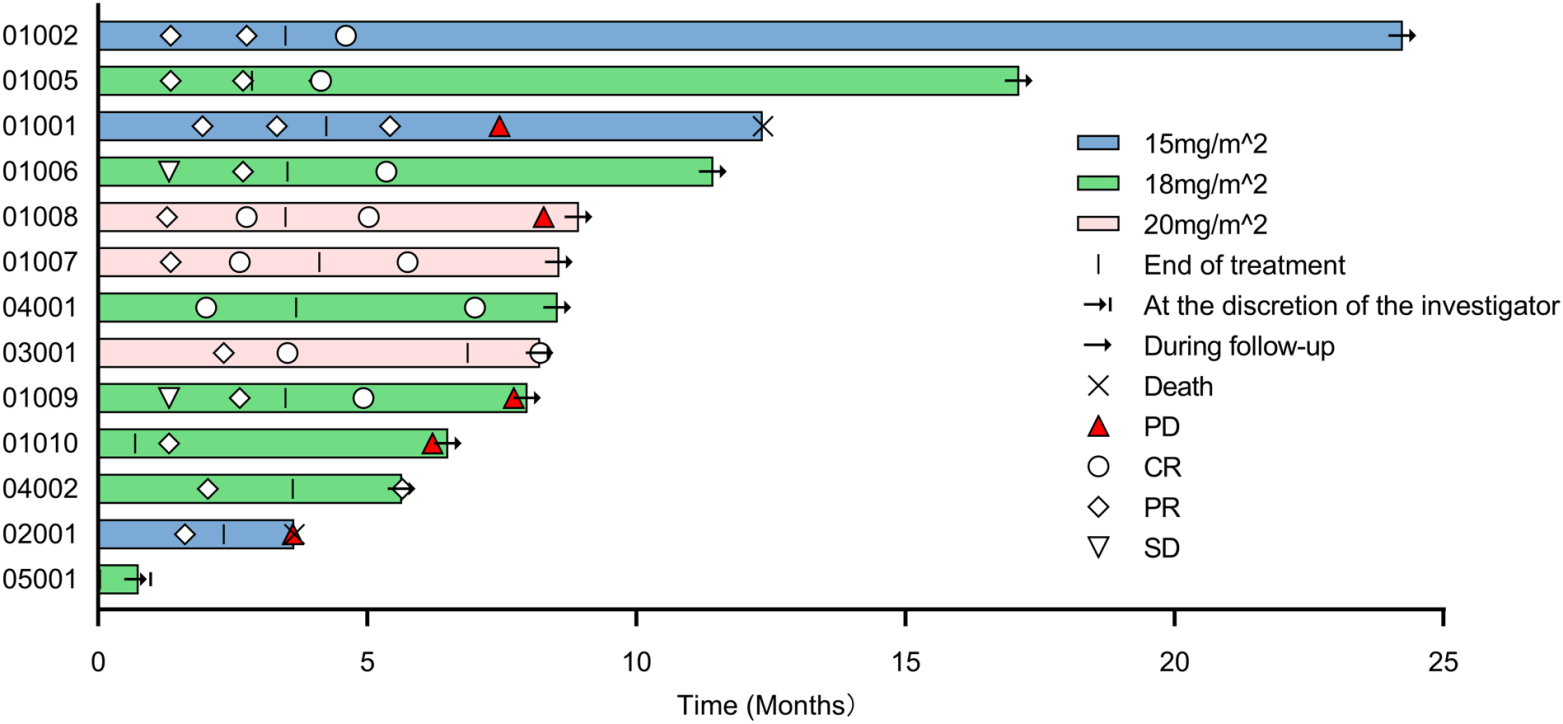
Time on treatment. PD, progressive disease; CR, complete response; PR, partial response; SD, stable disease.

**Figure 3:**
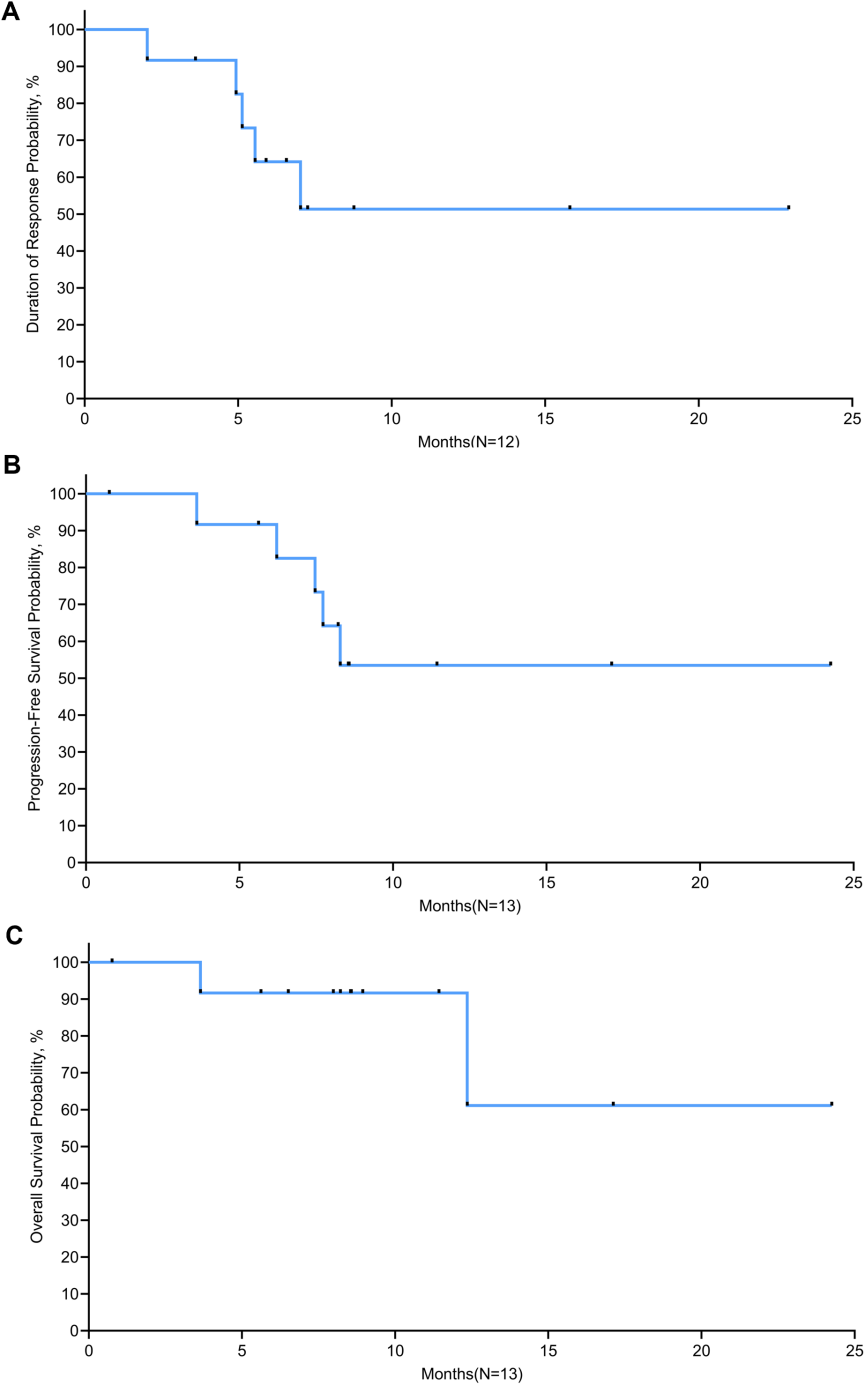
Kaplan-Meier curves of **(A)** duration of response, **(B)** progression-free survival, and **(C)** overall survival.

Tumor response was evaluated in 12 evaluable patients (**Table 3**). In the 15 mg/m^2^ cohort, the best response was observed in 3 patients, with 1 (33.3%) showing CR and 2 (66.7%) achieving PR, resulting in an ORR of 100% (95% CI, 29.2-100.0). In the 18 mg/m^2^ cohort, 6 patients exhibited an ORR of 100% (95% CI, 54.1-100.0), comprising 4 (66.7%) CR and 2(33.3%) PR. The 20 mg/m² cohort achieved an ORR of 100% (95% CI, 29.2-100.0) and a CR rate of 100%. Overall, the ORR for all patients was 100% (95% CI, 73.5-100.0), with 8 (66.7%) achieving CR and 4 (33.3%) PR. The time on treatment for all patients is shown in **Figure 2**. Five (38.5%) patients experienced progressive disease (PD) following the end of treatment, 2 in the 15 mg/m^2^ cohort, 2 in the 18 mg/m^2^ cohort, and 1 in the 20 mg/m^2^ cohort. Ten (76.9%) patients were continuing to undergo follow-up at the data cutoff. The median duration of response (DoR) was not reached, but responses were still ongoing in 7 (58.3%) of 12 responses, with the longest ongoing DoR of 22.9 months (**Figure 3A**).

**Table 3:** Summary of response data and anti-tumor activity outcomes.

Best overall response	15 mg/m^2^ (n=3)	18 mg/m^2^ (n=6)	20 mg/m^2^ (n=3)	Overall (n=12)
CR	1 (33.3)	4 (66.7)	3 (100.0)	8 (66.7)
95% CI	0.8-90.6	22.3-95.7	29.2-100.0	34.9-90.1
PR	2 (66.7)	2 (33.3)	0	4 (33.3)
SD	0	0	0	0
PD	0	0	0	0
ORR	100.0 (29.2-100.0)	100.0 (54.1-100.0)	100.0 (29.2-100.0)	100.0 (73.5-100.0)

Data are presented as n (%) or n (%, 95% CI).

CI, confidence interval; PD, progressive disease; CR, complete response; PR, partial response; SD, stable disease; ORR, overall response rate.

Survival was evaluated in 13 patients. The median follow-up time was 8.5 months at the data cutoff. Two (15.4%) patients died after disease progression. The median PFS was not reached, with PFS rates of 91.7% at 6 months and 53.5% at 12 months and plateau in the survival curve at 18 months (**Figure 3B**). The median OS was not reached; the 1-year and 18-month OS rates were 91.7% and 61.1%, respectively (**Figure 3C**).

## 4 Discussion

The first-line therapy for patients with untreated PTCL patients remains challenging given the limited choice of effective anti-tumor agents. To the best of our knowledge, this Phase I study represented the first demonstration of the tolerability of the CMOEP regimen, with hematologic toxicities as the predominant adverse events. Furthermore, this regimen exhibited promising anti-tumor activity, with an ORR of 100% and CR rate of 66.7% in untreated patients with PTCL.

None of the DLTs occurred in three dose cohorts, indicating that this CMOEP combination regimen had an acceptable safety profile in the untreated PTCL population. In addition, the reported hematological AEs were known and also common, such as neutrophil count decreased, white blood cell decreased, lymphocyte count decreased, anemia and platelet count decreased, similar to CHOP regimen, CHOP-like regimen or novel antineoplastic agent (denileukin diftitox [a genetically engineered fusion protein], azacitidine [an epigenetic modifier], and everolimus [an mTOR inhibitor]) plus CHOP regimen (21–24). This implied that most of these AEs mostly resulted from the chemotherapeutic agents alone and Lipo-MIT did not increase the risk of hematological toxicity. It is noteworthy that the previous phase I study conducted on the same population reported an AE incidence rate of 100% (20, 25). Slightly higher than the 92.3% observed with the current regimen, which may further underscore the safety profile of our regimen. Cardiac toxicity was monitored as an adverse event of special interest because anthracyclines are recognized risk factors for cardiotoxicity that compromise the survival of cancer patients (26). Although some patients inevitably experienced low-grade cardiac-related toxicity during the treatment, biomarkers of cardiac safety suggested that the current regimen has not resulted in myocardial injury or heart failure. Both LVEF and hs-cTnI were consistently within normal ranges throughout the treatment cycle. Additionally, there was no increase in the number of patients displaying abnormal levels of cTnT, cTnI, BNP, and NT-proBNP from baseline to the end of treatment. The above findings further demonstrated its favorable cardiac safety profile. This may be attributed to the special preparation process and targeted delivery mechanism of mitoxantrone hydrochloride liposome, which potentially mitigates harm to non-target organs like the heart compared to traditional anthracyclines (16, 27). In general, the CMOEP regimen was well-tolerated and without new unexpected safety signals. However, more toxicity evaluation in phase II is needed to refine further the real toxicity that can be expected with prolonged administration with this dose schedule.

Concerning efficacy, preliminary evidence of clinical activity was observed in this population. In all patients evaluated for the best response, an ORR of 100% and CR rate of 66.7% was observed. This outcome is particularly encouraging when compared to historical data from similar patient populations treated with other combination therapies, such as mitoxantrone hydrochloride liposome plus cyclophosphamide, vincristine, and prednisone (ORR, 84%; CR rate, 52%) (20), alemtuzumab (a monoclonal antibody) plus CHOP (ORR, 72%; CR rate, 60%) (28) and lenalidomide (an immunomodulatory drug) plus CHOEP (ORR, 87%; CR rate, 63%) (29), highlighting its potential as a more efficacious therapeutic strategy. Previous studies evaluating chemotherapy combined with targeted agents as first-line therapy for patients with PTCL have reported one-year OS rates of 67%-78.2% (14, 15, 23). Compared to these studies, the improvement of our regimen might be partly attributed to the younger age profile of our overall patient cohort (median age, 52 vs. 62-66 years old) and having a lower proportion of patients classified as high-intermediate or high-risk according to the IPI (7.7% vs. 43-61.1%). Furthermore, our follow-up was shorter than these studies (8.5 vs 20-72 months), and we reason that the OS benefit would be clearer as the follow-up continues. While the 6-month PFS rate observed in our study was higher than reported in previous research (91.7% vs. 78.2%) (20), the median PFS was not reached. This might be explained by the lower sample sizes, short follow-up time, and variations in patients’ backgrounds. Unfortunately, 2 patients died due to disease progression, but their deterioration was attributed to the natural course of the disease and was not related to the study medication. Despite the results seeming promising, the cross-trial comparisons should be made cautiously as clinical trials differ in patient selection, trial design, and other factors. Further validation through larger randomized controlled trials and long-term follow-up for the durability of responses would be necessary to confirm these preliminary findings and establish the new regimen’s true clinical benefit.

An obvious limitation of this study is its small sample size and not enough patients had experienced progression of disease at the time of data cutoff, which makes the time to the median of PFS not estimable for this population. As these data are from a non-randomized, open-label study, the data have some inherent limitations. Moreover, given the high heterogeneity of PTCL, factors such as different disease subtypes, stages, and ECOG performance status may affect our interpretation of the results. Despite all these limitations, larger and longer-term studies are warranted in the future to validate and broaden the applicability of the observed therapeutic effects of this regimen.

## 5 Conclusions

In summary, this phase I study successfully determined that the CMOEP regimen had a manageable safety profile, with RP2D of 18 mg/m^2^. Moreover, it showed promising anti-tumor activity, evidenced by ORR of 100% and CR rate of 66.7% as first-line therapy in patients with untreated PTCL. These encouraging outcomes have provided supportive evidence for the development of a phase II study (ClinicalTrials.gov identifier NCT06433362), which will delve deeper into the therapeutic potential of the CMOEP regimen as a first-line treatment for PTCL patients, thereby contributing to the expansion of efficacious treatment options in this challenging hematological malignancy.

## Data Availability

The original contributions presented in the study are included in the article/**Supplementary Material**. Further inquiries can be directed to the corresponding author.
