# Supplementary material for: CMOEP regimen in the treatment of untreated peripheral T-cell lymphoma: a multicenter, single-arm, phase I study
Source: Front Immunol. 2025 Apr 11;16:1551723. doi: 10.3389/fimmu.2025.1551723 (PMC12021869; doi:10.3389/fimmu.2025.1551723)
Supplement: Supplementary file 1 [file Table1.docx]

**Table S1 Complete eligibility criteria**

| **Inclusion criteria** |
| --- |
| All subjects participating in this clinical trial must meet all of the following criteria:   1. Subjects fully understand and voluntarily participate in this study and sign informed consent. 2. Age ≥18, ≤65 years, no gender limitation. 3. Expected survival ≥ 3 months. 4. Histologically confirmed diagnosis of Peripheral T-cell lymphoma: 5. Peripheral T-cell lymphoma unspecified (PTCL-NOS); 6. Angioimmunoblastic T-cell lymphoma (AITL); 7. Anaplastic large T-cell lymphoma (ALCL), ALK+; 8. Anaplastic large T-cell lymphoma (ALCL), ALK-; 9. Other subtypes of PTCL that the investigator think can be included in the group. 10. No previous treatment for PTCL, including chemotherapy, targeted therapy, immunotherapy, local radiotherapy for lymphoma (except for local radiotherapy to alleviate tumor related symptoms), surgical treatment. 11. Subjects must have at least one evaluable or measurable lesion per lugano2014 criteria: for lymph node lesions, the length and diameter should be > 1.5cm; For non-lymph node lesions, the length and diameter should be > 1.0cm. 12. Eastern Cooperative Oncology Group (ECOG) Performance Status (PS) 0-1. 13. The following baseline laboratory criteria are required: 14. Absolute neutrophil count (ANC) ≥ 1.5×10^9/L; 15. Platelet count (PLT) ≥ 75×10^9/L; 16. Hemoglobin (HB) ≥ 90 g/L. 17. Total Serum creatinine (Scr) ≤ 1.5×upper limit of normal (ULN), Alanine aminotransferase (ALT) and aspartate aminotransferase (AST) ≤ 2.5×ULN, bilirubin (TBIL) ≤ 1.5×ULN. |
| **Exclusion criteria** |
| An individual who meets any of the following criteria will be excluded from participation in this study:   1. The subject had previously received any of the following anti-tumor treatments: 2. Subjects who have been treated with mitoxantrone or mitoxantrone liposomes; 3. Previously received doxorubicin or other anthracycline treatment, and the total cumulative dose of doxorubicin was more than 360 mg/m^2^ (1 mg doxorubicin equivalent to 2 mg epirubicin). 4. Hypersensitivity to any study drug or its components. 5. Uncontrolled systemic diseases (such as active infection, uncontrolled hypertension, diabetes, etc.) 6. Heart function and disease meet one of the following conditions: 7. Long QTc syndrome or QTc interval > 480 ms; 8. Complete left bundle branch block, grade II or III atrioventricular block; 9. Serious and uncontrolled arrhythmias requiring drug treatment; 10. New York Heart Association grade ≥ II; 11. Cardiac ejection fraction (LVEF) < 50%; 12. A history of myocardial infarction, unstable angina pectoris, severe unstable ventricular arrhythmia or any other arrhythmia requiring treatment, a history of clinically serious pericardial disease, or ECG evidence of acute ischemia or active conduction system abnormalities within 6 months before recruitment. 13. Hepatitis B and hepatitis C active infection (defined as hepatitis B virus surface antigen positive and hepatitis B virus DNA higher than 1x10^3 copy/mL; hepatitis C virus RNA high than 1×10^3 copy/mL). 14. Human immunodeficiency virus (HIV) infection (HIV antibody positive). 15. Patients with other malignant tumors, except for effectively controlled non melanoma skin basal cell carcinoma, breast/cervical carcinoma in situ and other tumor during the past 5 years. 16. Patients with primary or secondary central nervous system (CNS) lymphoma or history of CNS lymphoma. 17. Pregnant and lactating women and patients of childbearing age who are unwilling to take contraceptive measures. 18. Unsuitable subjects for this study determined by the investigator. |

**Table S2 Treatment-related adverse events in the safety population**

| TRAEs, all grades | 15 mg/m^2^ | 18 mg/m^2^ | 20 mg/m^2^ | Total |
| --- | --- | --- | --- | --- |
|  | (n=3) | (n=7) | (n=3) | (n=13) |
| All TRAEs | 3 (100.0) | 6 (85.7) | 3 (100.0) | 12 (92.3) |
| Neutrophil count decreased | 3 (100.0) | 6 (85.7) | 3 (100.0) | 12 (92.3) |
| White blood cell decreased | 3 (100.0) | 6 (85.7) | 3 (100.0) | 12 (92.3) |
| Anemia | 2 (66.7) | 5 (71.4) | 3 (100.0) | 10 (76.9) |
| Lymphocyte count decreased | 3 (100.0) | 4 (57.1) | 2 (66.7) | 9 (69.2) |
| Platelet count decreased | 2 (66.7) | 4 (57.1) | 2 (66.7) | 8 (61.5) |
| Limb numbness | 1 (33.3) | 3 (42.9) | 3 (100.0) | 7 (53.8) |
| Alopecia | 1 (33.3) | 4 (57.1) | 2 (66.7) | 7 (53.8) |
| Fatigue | 1 (33.3) | 4 (57.1) | 1 (33.3) | 6 (46.2) |
| Fever | 1 (33.3) | 2 (28.6) | 1 (33.3) | 4 (30.8) |
| Hyperuricemia | 0 | 2 (28.6) | 2 (66.7) | 4 (30.8) |
| Anorexia | 0 | 2 (28.6) | 2 (66.7) | 4 (30.8) |
| Skin hyperpigmentation | 0 | 2 (28.6) | 2 (66.7) | 4 (30.8) |
| Dizziness | 0 | 1 (14.3) | 2 (66.7) | 3 (23.1) |
| Nausea | 0 | 1 (14.3) | 2 (66.7) | 3 (23.1) |
| Cough | 0 | 1 (14.3) | 2 (66.7) | 3 (23.1) |
| Productive cough | 0 | 1 (14.3) | 2 (66.7) | 3 (23.1) |
| Hypertriglyceridemia | 0 | 2 (28.6) | 1 (33.3) | 3 (23.1) |
| Hyperhidrosis | 0 | 1 (14.3) | 2 (66.7) | 3 (23.1) |
| Hyperglycemia | 1 (33.3) | 1 (14.3) | 0 | 2 (15.4) |
| Mucositis oral | 1 (33.3) | 1 (14.3) | 0 | 2 (15.4) |
| Vomiting | 0 | 1 (14.3) | 1 (33.3) | 2 (15.4) |
| Sore throat | 0 | 0 | 2 (66.7) | 2 (15.4) |
| Lung infection | 0 | 2 (28.6) | 0 | 2 (15.4) |
| Hypercholesterolemia | 0 | 2 (28.6) | 0 | 2 (15.4) |
| Lumbago | 0 | 1 (14.3) | 1 (33.3) | 2 (15.4) |
| Hypoalbuminemia | 0 | 2 (28.6) | 0 | 2 (15.4) |
| Epistaxis | 0 | 0 | 1 (33.3) | 1 (7.7) |
| Constipation | 0 | 1 (14.3) | 0 | 1 (7.7) |
| Infections | 1 (33.3) | 0 | 0 | 1 (7.7) |
| Hypocalcemia | 0 | 1 (14.3) | 0 | 1 (7.7) |
| Hiccups | 1 (33.3) | 0 | 0 | 1 (7.7) |
| Febrile neutropenia | 1 (33.3) | 0 | 0 | 1 (7.7) |
| Pneumonia | 0 | 1 (14.3) | 0 | 1 (7.7) |
| High triglycerides | 0 | 0 | 1 (33.3) | 1 (7.7) |
| Hyperphosphatemia | 1 (33.3) | 0 | 0 | 1 (7.7) |
| Body aches | 0 | 1 (14.3) | 0 | 1 (7.7) |
| Nail discoloration | 0 | 1 (14.3) | 0 | 1 (7.7) |
| Ankle swelling | 0 | 1 (14.3) | 0 | 1 (7.7) |
| Glucosuria | 1 (33.3) | 0 | 0 | 1 (7.7) |
| Rash | 1 (33.3) | 0 | 0 | 1 (7.7) |
| Insomnia | 1 (33.3) | 0 | 0 | 1 (7.7) |
| Gastrointestinal disorders | 1 (33.3) | 0 | 0 | 1 (7.7) |
| Gastrointestinal disorders-other | 1 (33.3) | 0 | 0 | 1 (7.7) |
| Palpitations | 0 | 1 (14.3) | 0 | 1 (7.7) |
| Blood bilirubin increased | 1 (33.3) | 0 | 0 | 1 (7.7) |
| Back/leg pain | 0 | 1 (14.3) | 0 | 1 (7.7) |
| Nail loss | 0 | 1 (14.3) | 0 | 1 (7.7) |
| Gamma-glutamyltransferase increased | 1 (33.3) | 0 | 0 | 1 (7.7) |
| Alanine aminotransferase increased | 1 (33.3) | 0 | 0 | 1 (7.7) |
| Flu like symptoms | 0 | 0 | 1 (33.3) | 1 (7.7) |
| Chest pain | 0 | 0 | 1 (33.3) | 1 (7.7) |
| Sinus arrhythmia | 0 | 1 (14.3) | 0 | 1 (7.7) |
| Palmar-plantar erythrodysesthesia syndrome | 1 (33.3) | 0 | 0 | 1 (7.7) |
| Electrocardiogram T wave abnormal | 1 (33.3) | 0 | 0 | 1 (7.7) |
| Ascending aorta widened | 1 (33.3) | 0 | 0 | 1 (7.7) |
| Urinary white blood cell count decreased | 0 | 1 (14.3) | 0 | 1 (7.7) |
| Pulmonary hypertension | 0 | 1 (14.3) | 0 | 1 (7.7) |
| Sinus tachycardia | 0 | 1 (14.3) | 0 | 1 (7.7) |
| Hematuria | 0 | 1 (14.3) | 0 | 1 (7.7) |

Data are n (%).

**Abbreviations:** TRAEs=treatment-related adverse events.

**Table S3 Biomarkers levels of cardiac safety before and after treatment**

|  | **Before treatment** | **After treatment** |
| --- | --- | --- |
| BNP, pg/mL |  |  |
| N (miss) | 11 (2) | 11 (2) |
| Median (min, max) | 10.0 (8.0,223.0) | 10.0 (10.0,59.9) |
| LVEF, % |  |  |
| N (miss) | 13 (0) | 12 (1) |
| Median (min, max) | 64.0 (60.0,70.0) | 64.0 (60.0,71.0) |
| hs-cTnI, pg/mL |  |  |
| N (miss) | 8 (5) | 8 (5) |
| Median (min, max) | 1.1 (0.3,7.1) | 1.7 (1.1,5.4) |

**Abbreviations:** BNP=brain natriuretic peptide; LVEF=left ventricular ejection fraction; hs-cTnI= high-sensitivity cardiac troponin I

**Figure S1 Biomarkers levels of cardiac safety throughout the treatment cycle***

*A 53-year-old male patient was diagnosed with monomorphic epitheliotropic intestinal T-cell lymphoma and classified as Ann Arbor stage IV. He received treatment with the CMOEP regimen for 0.76 months. During this period, his NT-proBNP levels increased from 615 pg/ml at the baseline (C1) to 668 pg/ml at the end of the treatment (End; Figure S1-F). However, the observed increase in NT-proBNP levels cannot be excluded as potentially being associated with the CMOEP regimen.


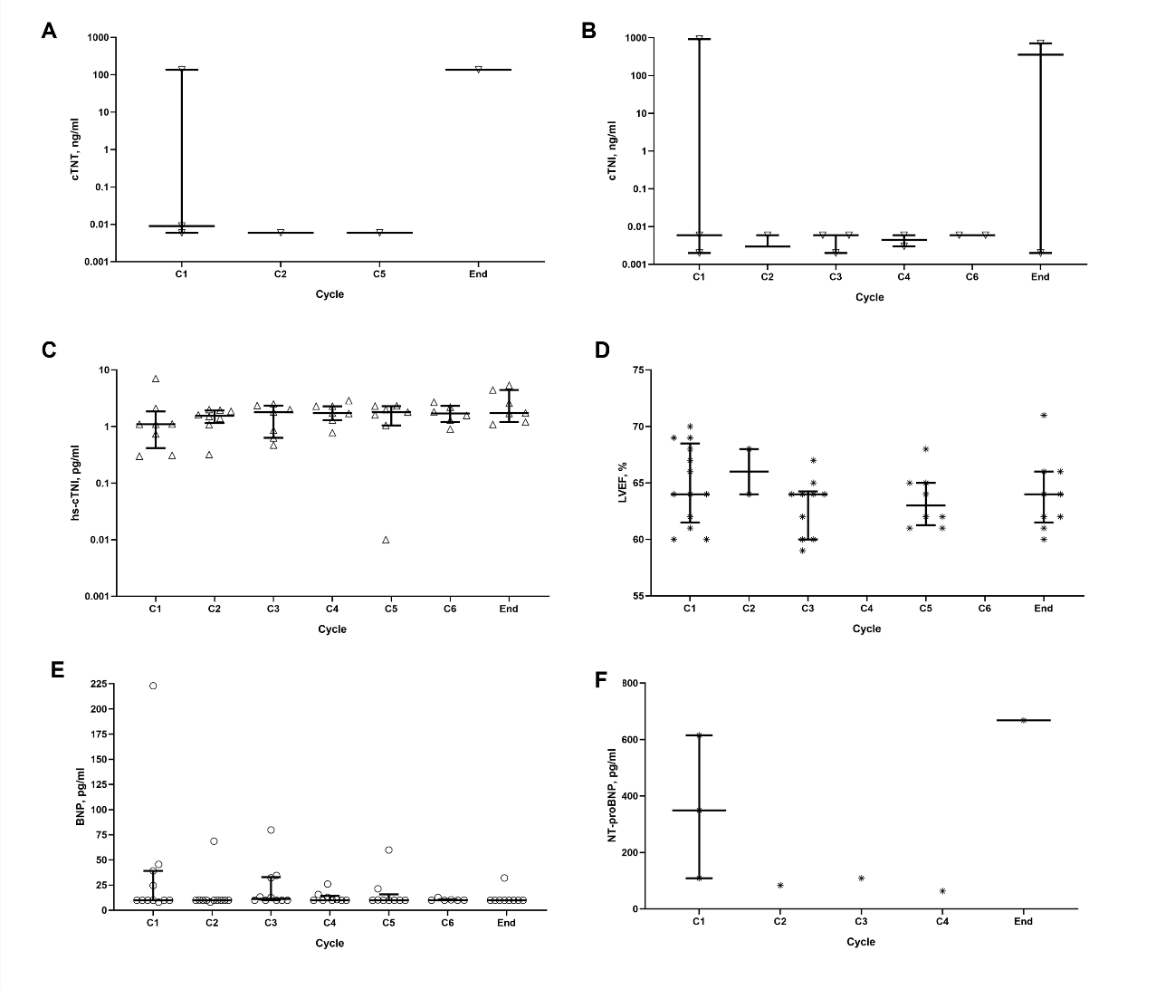


**Abbreviations:** cTNT=myocardial troponin T; cTNI=myocardial troponin I; hs-cTNI=high-sensitivity cardiac troponin I; LVEF=left ventricular ejection fraction; BNP= brain natriuretic peptide; NT-proBNP=N-terminal brain natriuretic peptide.
